# Mental health of healthcare workers during the Covid-19 pandemic: Analysis of the psychological problems faced by the healthcare workers over a period of three months during Covid-19 pandemic

**DOI:** 10.1192/j.eurpsy.2022.1390

**Published:** 2022-09-01

**Authors:** L. Spirkoska, M. Simonovska, O. Jankuloska, N. Ivceva, N. Olumcev

**Affiliations:** City General Hospital 8th September , Psychiatry, Skopje, North Macedonia

**Keywords:** Depression, Anxiety, covid, Insomnia

## Abstract

**Introduction:**

SARS-COV 2 virus and the disease caused by the virus is a challenge for the healthcare workers since the beggining of the Covid-19 pandemic. Working with huge number of patient who need hospital care at the same time but also having scare information about the virus resulted with physical exhaustion, increased workload and mostly fear among the healthcare workers.

**Objectives:**

The purpose of the study was to expand our knowledge about the mental health of the healthcare workers and explore most common psychological problems they faced during the Covid-19 pandemic over a period of three month.

**Methods:**

All participants in the study work in the same public hospital in Skopje, North Macedonia in one of the following job positions: physicians, nurses, paramedics, and hygienists. They anonymously filled out a google form answering questions about their mental health. Questions in the form were created based on Beck Depression Scale and the GAD-7 Scale for Anxiety along with questions about the gender, age, and job position.

**Results:**

The results we gained from this study are showing that the participants experienced insomnia, reduced concentration, intense feelings of restlessness and fatigue, less energy and mostly lack of job satisfaction.

**Conclusions:**

Those symptoms indicates that healthcare workers developed anxiety and depression while working with patient infected by the SARS-COV 2 virus.

**Disclosure:**

No significant relationships.

